# Long-term outcomes in radically treated synchronous vs. metachronous oligometastatic non-small-cell lung cancer

**DOI:** 10.1186/s12885-016-2379-x

**Published:** 2016-06-02

**Authors:** Jochen Fleckenstein, Alev Petroff, Hans-Joachim Schäfers, Thomas Wehler, Jakob Schöpe, Christian Rübe

**Affiliations:** Department of Radiotherapy and Radiation Oncology, Saarland University Medical Center, Homburg, Germany; Department of Thoracic and Cardiovascular Surgery, Saarland University Medical Center, Homburg, Germany; Department of Respiratory and Critical Care Medicine, Saarland University Medical Center, Homburg, Germany; Institute for Medical Biometry, Epidemiology and Medical Informatics, Saarland University, Campus Homburg, Saarbrücken, Germany

**Keywords:** NSCLC, Oligometastatic disease, Radical treatment, SBRT

## Abstract

**Background:**

Radical treatment for oligometastatic non-small-cell lung cancer (NSCLC) has a curative potential for selected patients. The present retrospective study was designed to examine the relevance of synchronous vs. metachronous manifestations as a potential prognostic factor when ablative treatments are performed in oligometastatic disease.

**Methods:**

Seventy-five patients with radically treated oligometastatic NSCLC were identified, of whom 39 presented with synchronous and 36 with metachronous metastatic manifestations. For patients with synchronous metastases, an additional therapy of the thoracic locoregional disease with a curative intent (either surgery or radiochemotherapy) was required. All patients with metachronous metastases had a documented remission of the primary tumor. Ablative treatment of the complete extent of oligometastatic disease consisted (as a minimum requirement) of either complete surgical resection or definitive ablative stereotactic radiotherapy. A comparative survival analysis of two groups of patients with oligometastatic NSCLC (synchronous vs. metachronous) and a complementary analysis of prognostic factors for the whole group of patients (by means of Cox regression analysis) was performed. Endpoints were median overall and progression-free survival (OS, PFS, respectively).

**Results:**

Of the 75 patients, 57 presented with a solitary metastasis, in only 7 patients metastastatic lesions were present in ≥2 organs and 66 patients had a Karnofsky performance score (KPS) of 80 % or 90 %. The median follow-up was 54.0 months (95 % CI 28–81), the median OS 21.8 months (16.1–27.6) and the median PFS 13.7 months (9.7–17.6). In univariable Cox regression analysis, no single clinical factor was significantly associated with OS. For PFS both ‘metastatic involvement of ≥2 organs vs. 1 organ’ (hazard ratio (HR) 0.43, 0.23–0.83, *p* = 0.012) and a ‘KPS of 90 % vs. 70–80 %’ (HR 4.32, 1.73–10.89, *p* = 0.02) were significant prognostic factors as calculated by multivariable analysis. Comparing the cohorts with synchronous (*n* = 39) vs. metachronous oligometastases (*n* = 36), no differences in median OS and PFS were found. Both cohorts were well-balanced except for the KPS, which was significantly superior in patients with synchronous oligometastases.

**Conclusions:**

Radical treatment of oligometastatic NSCLC was associated with acceptable long-term survival rates in patients with good KPS and it was equally effective for synchronous and metachronous manifestations.

**Electronic supplementary material:**

The online version of this article (doi:10.1186/s12885-016-2379-x) contains supplementary material, which is available to authorized users.

## Background

Since its introduction by Hellman and Weichselbaum in 1995 [1] the concept of ‘oligometastatic disease’ has gained clinical relevance [2]. Meanwhile, most metastatic lesions inside or outside the brain are amenable to an increasing variety of ablative therapeutic modalities, be it surgery, stereotactic ablative (intra- or extracranial) radiotherapy, or radiofrequency or microwave ablation. For non-small-cell lung cancer (NSCLC) – as is the case for most entities of malign solid tumors – the state of oligometastatic disease has not yet been adopted as a discrete, single category in the AJCC staging system. Nevertheless, aggressive local treatment for limited site oligometastic disease is recommended in the current version of NCCN-guidelines, where it is yet confined to brain and adrenal involvement (http://www.nccn.org/professionals/physician_gls/pdf/nscl.pdf). From the large number of retrospective trials it may be inferred that, at least in specialized centers, radical treatment approaches for oligometastases are widely used. A recent metaanalysis of aggressive treatment of oligometastatic NSCLC based on 757 individual patient data revealed several prognostic factors [3]. Among other determinants, a significantly worse outcome in overall survival for synchronous as opposed to metachronous appearance of oligometastases was reported in that metaanalysis. This was especially the case for patients with concurrent N1/N2 disease. Thus, the role of curative approaches in synchronous oligometastatic NSCLC seems to be questionable.

The presented retrospective analysis of a group of 75 patients with oligometastatic NSCLC was designed to examine potential predictive factors for long-term survival outcome of radical treatments (including innovative contemporary irradiation technology) in terms of OS and PFS and as an in-depth analysis opposing synchronous to metachronous metastases.

## Methods

### Study population and inclusion criteria

The patient database of the Department for Radiotherapy and Radiation Oncology, Saarland University Medical Center, was screened for patients with histologically confirmed NSCLC who had presented with oligometastatic disease at the time of initial diagnosis (‘synchronous’) or had oligometastatic recurrence during the course of disease at least three months after the initial diagnosis (‘metachronous’). Only patients with ≤5 metastases (≤3 brain metastases) were included and radical treatment for all metastases was mandatory, i.e. either complete resection of the metastastic lesion ± adjuvant radiotherapy or definitive radiotherapy to the metastatic lesion with a biologically effective dose for α/β = 10 Gy (BED10) of ≥60 Gy. Additionally, the thoracic locoregional tumor in patients with synchronous manifestations had to be treated with a curative scheme according to the current standard of care (either complete resection with or withouth adjuvant/neoadjuvant chemotherapy/radiotherapy or definitive concurrent radiochemotherapy with at least 60 Gy, conventional fractionation, and a platinum-based chemotherapy). For the cohort with metachronous metastases, a controlled locoregional disease was required as documented by a computed tomography of the chest. Seventy-five patients were identified, who met all prerequisites and received treatment between March 2000 and April 2015 (55 of these patients (73 %) received treatment between 2008 and 2015).

Primary endpoints were overall survival (OS) and progression-free survival (PFS). For patients with synchronous metastases, survival data were calculated from the day of first treatment of either the primary tumor or metastatic lesions and for patients with metachronic metastases from the day of first treatment of oligometastatic disease.

### Statistical analysis

Differences in baseline characteristics between synchronous and metachronous oligometastatic disease groups regarding categorical variables were assessed using the chi-squared test or Fisher’s exact test (for 2x2 tables)/Fisher-Freeman-Halton test (for RxC tables). Statistical differences concerning continuous variables were examined using the two-sample t-test (for normal distributions). All applied statistical tests were two-sided, and a p-value less than 0.05 was considered statistically significant. Univariable Cox proportional hazard models were performed to assess predictive factors associated with OS and PFS. Finally, statistically significant predictors from univariable analyses were used in multivariable analyses with a forward selection procedure. As predictor variables we selected age (>60 vs. ≤ 60 years); sex; KPS (90 % vs. 70–80 %); weight loss (≤5 % vs. >5 %); ‘metachronous’ vs. ‘synchronous’ oligometastases; histology (squamous vs. adenocarcinoma); T-stage (T3/T4 vs. T1/T2); N-Stage (N2/3 vs. N0/N1); Thoracic UICC stage (III vs. II/I); localization of metastases (other vs. brain); number of metastases (≥2 vs. 1); number of involved organs (≥2 vs. 1); treatment of primary tumor (without vs. with surgery); treatment of oligometastases (radiotherapy vs. surgery); FDG-PET based staging; additional chemotherapy.

Kaplan-Meier estimates were calculated for (median) OS and (median) PFS. Differences between strata were calculated by use of log-rank test. All statistical tests were performed with SPSS statistics version 23 (IBM Corporation, Armonk, NY).

## Results

The patient characteristics are presented in Table 1. The included patients had predominantly favorable baseline criteria: the majority of patients (88 %) had a good Karnofsky performance score (KPS) of 80 % or 90 % and most patients suffered no weight loss prior to the analyzed treatment (87 %). Oligometastatic disease was mostly limited: 91 % of patients had metastases confined to one involved organ and 76 % of patients presented with a solitary metastasis. Brain metastases outweighed all other involved metastastic sites. Three patients with adenocarcinoma histology had a mutation of the epidermal growth factor receptor and received additional treatment with erlotinib. Patients were additionally stratified according to either synchronous or metachronous occurrence of oligometastases. Both groups were well balanced with respect to most clinical criteria (the only exemption was KPS, which was significantly superior in patients with synchronous occurrence of metastases). For patients with metachronous metastases, the median interval between the first diagnosis of NSCLC and detection of oligometastatic disease was 15.2 months (range 3–58 months), which may diminish the impact of the modality of the earlier treatment of the primary tumor in this subgroup.

**Table 1:** Patient characteristics

	All	Synchronous	Metachronous	*p*
Total no. of patients	75	39	36	
Age, years				
mean ± SD	59.1 ± 8,4	59.5 ± 9,1	58.5 ± 7,6	0.63^b^
range	39-77	39-77	42-77	
Sex, no. (%)				
Male	43 (57)	23 (59)	20 (56)	0.76^c^
Female	32 (43)	16 (41)	16 (44)	
KPS, no. (%)				
70	9 (12)	3 (8)	6 (17)	
80	38 (51)	14 (36)	24 (66)	0.002^d^
90	28 (37)	22 (56)	6 (17)	
Weight loss, no. (%)				
<5%	65 (87)	36 (92)	29 (81)	0.18^e^
>5%	10 (13)	3 (8)	7 (19)	
Histology				
Adenocarcinoma	50 (67)	26 (67)	24 (66)	
Squamous	17 (23)	9 (23)	8 (22)	1.0^d^
Other	8 (10)	4 (10)	4 (11)	
T-Stage^a^, no. (%)				
T1	11 (15)	3 (8)	8 (22)	
T2	37 (50)	21 (54)	17 (47)	0.36^c^
T3	12 (16)	7 (18)	5 (14)	
T4	14 (19)	8 (20)	6 (17)	
N-Stage^a^, no. (%)				
N0	28 (38)	10 (25)	19 (53)	
N1	14 (19)	7 (18)	7 (19)	0.06^d^
N2	28 (38)	19 (49)	9 (25)	
N3	4 (5)	3 (8)	1 (3)	
Thoracic UICC stage, no. (%)				
IA	4 (5)	1 (3)	3 (8)	
IB	7 (9)	4 (10)	3 (8)	
IIA	14 (19)	6 (15)	9 (25)	0.08^d^
IIB	10 (14)	2 (5)	8 (22)	
IIIA	25 (34)	17 (44)	8 (22)	
IIIB	14 (19)	9 (23)	5 (14)	
Localization of metastases, no. (%)				
Brain	51 (62)	26 (59)	25 (65)	
Bone	7 (8)	5 (11)	2 (5)	
Lung	9 (11)	5 (11)	4 (11)	
Lymphatic	5 (6)	4 (9)	1 (3)	0.16^d^
Adrenal Gland	3 (4)	3 (7)	0	
Soft tissue	3 (4)	0	3 (8)	
Other	4 (5)	1 (2)	3 (8)	
No. of metastases, no. (%)				
1	57 (76)	29 (74)	28 (78)	
2	11 (15)	6 (15)	5 (14)	1.0^d^
3–5	7 (10)	4 (10)	3 (8)	
No. of involved organs, no. (%)				
1	68 (91)	34 (87)	34 (95)	0.43^e^
> 2	7 (9)	5 (13)	2 (5)	
Treatment of primary tumor, no. (%)				
Surgery ± (neo)adjuvant RT and/or CT	55 (73)	26 (66)	29 (80)	
RCT	18 (24)	12 (31)	6 (17)	0.34^d^
SBRT	2 (3)	1 (3)	1 (3)	
Treatment of oligometastases, no. (%)				
Surgery	12 (14)	7 (16)	5 (13)	
SRS/hSRT/SBRT	19 (23)	10 (22)	9 (23)	0.88^d^
Surgery/RT combined	49 (58)	25 (56)	24 (61)	
cf-RT	4 (5)	3 (7)	1 (3)	
FDG-PET based staging, no. (%)	51 (68)	34 (87)	17 (47)	
Additional chemotherapy, no. (%)	48 (64)	27 (69)	21 (58)	
FEV1, % predicted				
mean ± SD	76 ± 24	77 ± 27	74 ± 20	0.20^b^

*SD* standard deviation, *RT* radiotherapy, *CT* chemotherapy, *RCT* radiochemotherapy, *SBRT* stereotactic body radiotherapy, *SRS* stereotactic radiosurgery, *hSRT* hypofractionated stereotactic radiosurgery, *cf-RT* conventionally fractionated radiotherapy, *KPS* Karnofsky Performance Score, *FEV1* Forced expiratory volume during 1^st^ second of breathing maneuver

^a^refers to initial staging also for patients with metachronous metastases

^b^f-test for unpaired samples was used

^c^chi-square test was used

^d^Fisher-Freeman-Halton test was used

^e^Fisher's exact test was used

**Fig. 1:**
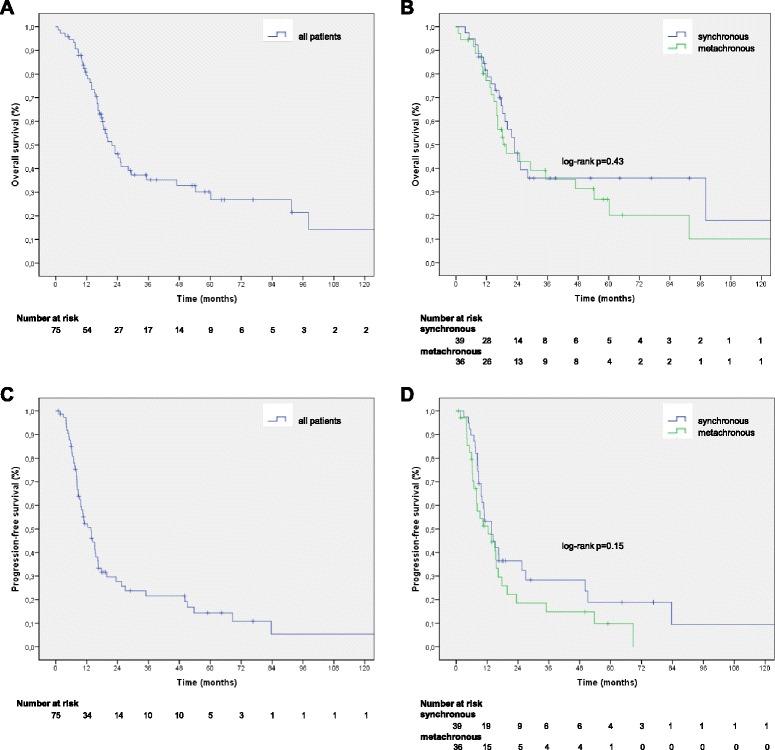
Overall survival (**a**) and progression-free survival (**c**) for all patients and stratified by synchronous vs. metachronous occurrence of oligometastic disease (**b**, **d**). The vertical markings indicate censored patients. The x-axis is capped at 121 months follow-up in all graphs, as in all groups two or less patients underwent a follow-up exceeding the presented interval

As to survival analysis, the median follow-up of all patients was 54.0 months (interquartile range (IQR) 52.5), the median follow-up for patients with synchronous oligometastases was 39.1 months (IQR 52.5) and for patients with metachronous oligometastaes 59.9 months (IQR 131.9), respectively. An actual number of 8 patients survived for more than 5 years and 47 patients have died during the examined follow-up period. The median OS for all patients was 21.8 months (95 % CI 16.1–27.6) and the PFS was 13.7 months (9.7–17.6) (Fig. 1). The actuarial 1-year-, 2-year- and 5 year-OS rates were 78 % , 44 % and 27 % 6 % with standard errors of 5 %, 6 % and 6 %, respectively. The corresponding 1-year-, 2-year- and 5 year-PFS rates were 50 % , 26 % and 11 % with standard errors of 5 %, 6 % and 6 %, respectively. For a variety of clinical factors, which may impact OS and PFS a univariable cox regression analysis was performed (Table 2). Most importantly, no statistically significant differences – with and without adjustment for KPS – between patients with synchronous and metachronous occurrence of oligometastases were observed concerning OS (*p* = 0.43) or PFS (*p* = 0.15; see Fig. 1). Also, none of the other examined factors were associated with OS in univariable analysis. However, three variables had a significant impact on PFS in univariable analysis: first, KPS, second, the presence of brain involvement and third, the limitation of oligometastatic spread to one organ. These three factors were subsequently examined via multivariable cox regression analysis. Here, a KPS of 90 % vs. 70–80 % (hazard ratio [HR] 0.43, 0.23–0.83, *p* = 0.012) and the metastastic involvement of more than one organ (HR 4.32, 1.73 – 10.89, *p* = 0.02) remained significant, whereas the presence of brain involvement did not.

**Table 2:** Univariable cox regression analysis of clinical factors potentially associated with overall survival (OS) and progression free survival (PFS)

	OS		PFS	
	Univariable HR (95 % CI)	*p*	Univariable HR (95 % CI)	*p*
Age, years				
>60 vs. S 60	1.25 (0,70-2,23)	0.44	1.10 (0,65-1,85)	0.72
Sex				
Female vs. male	1.19 0.66-2.13)	0.57	1.00 (0.59-1.69)	0.99
KPS				
90 % vs. 70–80%	0.68 (0.37-1.24)	0.21	0.51 (0.29-0.89)	0.018
Weight loss				
S 5 % vs. > 5 %	0.78 (0.31-1.99)	0.60	1.18 (0.53-2.63)	0.69
M1-stage - time of occurence				
Metachronous vs. synchronous	1.26 (0.71-2.24)	0.43	1.46 (0.87-2.48)	0.15
Histology				
Squamous vs.Adeno.	0.64 (0.28-1.46)	0.29	0.75 (0.37-1.50)	0.42
T -Stage^a^				
T3/T4 vs. T1/T2	0.79 (0.41-1.52)	0.29	1.02 (0.59-1.79)	0.94
N-Stage^a^				
N2/N3 vs.N0/N1	1.27 (0.71-2.25)	0.43	1.26 (0.74-2.15)	0.40
Thoracic UICC stage				
II vs. I	2.28 (0.87-5.96)	0.09	3.80 (1.36-10.69)	0.011
III vs. I/II	0.85 (0.48-1.51)	0.59	0.84 (0.49-1.43)	0.53
Localization of metastases				
Other vs. Brain	0.80 (0.43-1.51)	0.50	0.48 (0.26-0.91)	0.024
No. of metastases				
> 2 vs. 1	1.10 (0.56-2.17)	0.79	1.26 (0.71-2.26)	0.43
No. of involved organs				
> 2 vs. 1	1.03 (0.32-3.33)	0.96	2.90 (1.30-6.46)	0.009
T reatment of primary tumor				
Without vs. with Surgery	1.58 (0.74-3.40)	0.24	1.07 (0.58-1.96)	0.84
T reatment of oligometastases				
SRS/hSRT/SBRT vs. Surgery	1.00 (0.36-2.84)	0.99	0.75 (0.29-1.95)	0.55
FDG-PET based staging				
PET vs. no PET	1.00 (0.54-1.84)	1.00	0.84 (0.48-1.47)	0.54
Additional chemotherapy				
CT vs. no CT	0.69 (0.39-1.23)	0.21	0.84 (0.49-1.44)	0.52

*SD* standard deviation, *RT* radiotherapy, *CT* chemotherapy, *RCT* radiochemotherapy, *SBRT* stereotactic body radiotherapy, *SRS* stereotactic radiosurgery, *hSRT* hypofractionated stereotactic radiosurgery, *cf-RT* conventionally fractionated radiotherapy, *KPS* Karnofsky Performance Score

^a^refers to initial staging also for patients with metachronous metastases

The examined patients received a diversity of radical treatment schemes for oligometastases. Sixty-three out of 75 patients received radiotherapy, but only 23 patients received radiotherapy alone to metastatic sites. A majority of 49 patients underwent a combination of surgery and radiotherapy, of whom 37 patients had either whole brain radiotherapy or stereotactic radiotherapy as adjuvant treatment after resection of brain metastases.

Raw data of the patients’ characteristics and survival are included as an additional file (Additional file 1: raw_data).

## Discussion

The favorable survival data of the analyzed group of 75 patients (median OS 21.8 months) support the assumption that oligometastatic NSCLC constitutes a category of its own. In accordance with other retrospective analyses of radical treatment of oligometastic NSCLC [3] we found that this aggressive approach was mainly pursued in patients who had a good KPS and who were 10 years younger than the average German patient at the time of first diagnosis of lung cancer [4]. Interestingly, median OS of oligometastatic disease in these selected patients does not seem to be inferior to that of patients with inoperable stage III NSCLC, in whom it averages 21 months [5]. A multitude of single factors may influence the efficacy of radical treatment in oligometastatic disease and therefore survival. A recently published metaanalysis of 757 patients (the data were mostly derived from non-randomized retrospective trials) revealed synchronous (vs. metachronous) metastases, N1 or N2-stage (vs. N0) and ‘non-adenocarcinoma’ as being predictive for a decreased OS (as calculated in multivariable analyses). For patients with synchronous oligometastases the categories ‘surgery for the primary lung tumor’ and ‘smaller radiotherapy planning target volume’ were found to be predictive for improved OS in the analysis of Griffioen et al. [6], whereas the absence of nodal mediastinal involvement, adenocarcinoma, tumor size of <3 cm and age of <59 years were associated with improved OS in the study of Mordant et al. [7].

In the present study we specifically address the relevance of synchronous vs. metachronous metastases, because a thorough baseline stratification according to this variable had not been reported beforehand. The category ‘synchronous vs. metachronous’ discriminates two vastly different clinical scenarios: in patients with synchronous oligometastatic NSCLC most often a significant tumor burden is present and treatment of the metastatic site and multimodal treatment of the primary tumor have to be integrated in a reasonable manner. In comparison, treatment of patients with metachronous oligometastases and lasting local tumor control is confined to ablative therapy of the metastatic site and usually second line chemotherapy, whereas the applied initial therapy may generally limit therapeutic options. Considering this backdrop, the two groups of the present analysis were well balanced with respect to all baseline and treatment criteria (even though significant, the differences in KPS were small by an absolute measure). As we could not demonstrate significant differences in OS and PFS between synchronous and metachronous oligometastatic disease and no relevant selection bias was present between these groups, both of them seem equally qualified for radical treatment strategies.

It can be assumed that for patients with synchronous metastases local control of the primary tumor is at least as important for long-term survival as control of metastatic sites. The OS of patients with synchronous oligometastic disease compares favorably to the results reported in the Dutch prospective phase II trial [8]. In that trial, 39 patients with synchronous oligometastatic NSCLC received radical treatment, but all of these patients had definitive radio(chemotherapy) as the treatment of the primary tumor (in the present study 73 % of patients underwent thoracic surgery). Although only two patients had a local recurrence, the median overall survival (OS) was only 13.5 months (7.6–19.4). Guerra et al. reported a series of 78 patients with oligometastatic NSCLC who received chemoradiation to the primary site, but only 44 patients received additional radical local treatment of the metastases [9]. On multivariable analysis, they found an improved OS for patients, who were treated with 63 Gy or more of radiation to the primary site, who had a KPS >80 %, and who exhibited smaller tumor volumes, whereas whether or not oligometastatic disease was radically treated did only impact OS for the subset of patients with brain metastases. Su et al. also reported a survival benefit with radiotherapy of >63 Gy to the primary tumor in 93 patients with stage IV NSCLC, many of whom had multiple metastases [10].

Iyengar et al. reported favorable results for 24 patients with stage IV NSCLC and predominantly extracranial metastases, who experienced disease progression after first-line chemotherapy and were subsequently treated with stereotactic body radiation therapy (SBRT) to all involved sites in addition to erlotinib [11]. From the initiation of salvage SBRT + erlotinib median PFS was 14.7 months and median OS 20.4 months for these patients.

Due to the lack of prospective randomized studies, the added benefit of radical treatment of oligometastases remains unclear, although the strategy is supported by favorable survival data in comparison to historic data.

The sample size of the presented analysis is small from a statistical perspective, even though, with 75 analyzed patients, we could gather the second largest monoinstitutional data pool in comparison with the recent metaanalysis [3]. Certainly, the present study has some further limitations: first, the analyzed group of patients is inhomogeneous with respect to locoregional tumor extent, the involvement of metastatic sites, the number of metastases and the applied treatments; second, it is retrospective and therefore may contain inaccuracies with respect to the documentation of tumor progression dates (with a proportion of patients who needed to be censored in PFS survival analysis); and third, FDG-PET-staging was more frequently used in patients with synchronous vs. metachronous disease (87 % vs. 47 %), which may have impacted staging accuracy in the latter group. The majority of patients had brain metastases (62 %). Even though no impact of the involved metastatic site on survival was found, the number of examined patients is too small to perform a reliable comparison between different sites.

## Conclusions

The presented favorable survival data both in synchronous and metachronous oligometastatic NSCLC encourage the use of an aggressive, ablative therapy for selected patients. The actual benefit of radical therapy of metastatic sites has yet to evaluated in prospective randomized trials. Synchronous vs. metachronous manifestation of metastases was not of prognostic relevance in the examined cohort.

## Abbreviations

AJCC, American Joint Committee on Cancer; BED, biologically effective dose; CI, confidence interval; FDG-PET, fluoro-deoxy-D-glucose positron emission tomography; HR, hazard ratio; IQR, interquartile range; KPS, Karnofsky performance status; NCCN, National Comprehensive Cancer Network; NSCLC, non-small-cell lung cancer; OS, overall survival; PFS, progression-free survival; SBRT, stereotactic body radiotherapy; UICC, Union for International Cancer Control

## Additional file

Additional file 1: Raw_data of patients’ characteristics and survival. (XLSX 17 kb)
